# DC and AC Conductivity, Biosolubility and Thermal Properties of Mg-Doped Na_2_O–CaO–P_2_O_5_ Glasses

**DOI:** 10.3390/ma14102626

**Published:** 2021-05-17

**Authors:** Natalia Anna Wójcik, Sharafat Ali, Jakub Lech Karczewski, Bo Jonson, Michał Bartmański, Ryszard Jan Barczyński

**Affiliations:** 1 Advanced Materials Center, Gdańsk University of Technology, ul. Narutowicza 11/12, 80-233 Gdańsk, Poland; jakub.karczewski@pg.edu.pl (J.L.K.); michal.bartmanski@pg.edu.pl (M.B.); ryszard.barczynski@pg.edu.pl (R.J.B.); 2 Institute of Nanotechnology and Materials Engeenering, Faculty of Applied Physics and Mathematics, Gdańsk University of Technology, Narutowicza Street 11/12, 80-233 Gdańsk, Poland; 3 Department of Built Environment and Energy Technology, Linnaeus University, 35195 Växjö, Sweden; sharafat.ali@lnu.se (S.A.); bo.jonson@lnu.se (B.J.); 4 Faculty of Mechanical Engineering and Ship Technology, Gdańsk University of Technology, Narutowicza Street 11/12, 80-233 Gdańsk, Poland

**Keywords:** calcium–phosphate glass, FTIR, impedance spectroscopy, biosolubility, thermal properties

## Abstract

Bioactive glasses have recently been extensively used to replace, regenerate, and repair hard tissues in the human body because of their ability to bond with living tissue. In this work, the effects of replacing Na_2_O with MgO on the electrical, biosolubility, and thermal properties of the target glass 10Na_2_O–60P_2_O_5_–30CaO (in mol%) were investigated. The electrical properties of the glasses were studied with the impedance spectroscopy technique. At 473 K, DC conductivity values decreased from 4.21 × 10^−11^ to 4.21 × 10^−12^ S cm^−1^ after complete substitution of MgO for Na_2_O. All samples had a similar activation energy of the DC conduction process ~1.27 eV. Conduction mechanisms were found to be due to hop of ions: Na^+^, Mg^2+^_,_ and probable H^+^. FTIR analysis showed that, as the Mg content increased, the Q^2^ unit (PO_2_^−^) shifted towards higher wavenumbers. The proportion of Q^3^ unit (P_2_O_5_) decreased in the glass structure. This confirmed that the replacement of Na^+^ by Mg^2+^ was accompanied by concurrent polymerization of the calcium–phosphate glass network. The biosolubility test in the phosphate-buffered saline solution showed that the magnesium addition enhanced the biosolubility properties of Na_2_O–CaO–P_2_O_5_ glasses by increasing their dissolution rate and supporting forming CaP-rich layers on the surface. The glass transition temperature increased, and thermal stability decreased substantially upon substitution of Na_2_O by MgO.

## 1. Introduction

There has been great interest from scientific and technological points of view in phosphate glasses in recent years due to their potential applications as bioactive glasses [1]. Phosphate base glasses are also well-known for their low melting point and relatively high electrical conductivity [2]. The addition of different modifiers, such as Na, Ca, and Mg, has a significant influence on the structure, biodegradability and thermal properties of these glasses [3]. For example, Mg addition increases the glass transition temperature *T_g_* of phosphate glass caused by the high field strength of Mg^2+^ ions [4,5,6,7,8,9,10,11]. On the other hand, CaO–P_2_O_5_ [12] and Na_2_O–CaO–P_2_O_5_ [1] glass systems exhibit poor durability, high hygroscopic properties and a relatively fast dissolution in simulated body fluids (SBF). The biosolubility properties of Na_2_O–CaO–P_2_O_5_ can be modified by adding Mg. Lee et al. [4] studied the biodegradability properties of Na_2_O–CaO–MgO–P_2_O_5_ glasses and concluded that increased MgO content decreased the glass systems’ degradation rate. Regi et al. [13] reported the effect of adding MgO addition on the bioactive properties of CaO–P_2_O_5_-doped silicate glasses prepared by the sol–gel technique. It was observed that adding MgO slows down the rate of formation of the hydroxyapatite layer on the glass surface and increases the thickness of this layer when exposed to physiological solutions. Catauro et al. [14] prepared SiO_2_/ZrO_2_ composites with the same technique and studied their structure, drug absorption, bioactive and antibacterial properties. Catauro found that these materials can be considered carriers in the adsorption of an active drug.

The electrical properties of Na_2_O-containing phosphate glass systems are well-known [15,16,17,18]. In contrast, less literature is available about the dielectric properties of phosphate glasses doped with MgO [19,20]. Higazy et al. [20] measured the electrical properties of MgO–P_2_O_5_ glass systems and observed increased conductivity with increased MgO. They conclude that forming mobile Mg^2+^ ions is responsible for the ion conduction process in the glass network. Khor et al. [21] showed that dielectric permittivity, dielectric strength and DC conductivity decrease with increased magnesium oxide content in the ZnO–MgO–P_2_O_5_ system. This might be due to the dual behavior of Mg^2+^ that can act as a glass-former and/or modifier. Therefore, it is interesting to study the conduction processes in Na_2_O–CaO–MgO–P_2_O_5_ bioactive glasses to explain the mobility of ions [22,23,24,25]. New trends in bioresearch concern scaffolds prepared from bioglass composites, electrical conductors and can electrically stimulate cell growth [22,23]. Studies have shown that osteobonding and bone growth on the surface of hydroxylapatite (HPA) can be improved by generating a permanent surface charge on material [24,25].

This work aims to present in detail the influence of substitution of MgO for Na_2_O on the structure, electrical, biosolubility and thermal properties of calcium–phosphate glasses with a higher content of P_2_O_5_ and lower content of Na_2_O than the previously studied bioglasses [4]. Except in the case of glass stability, all properties display a pronounced dependency on MgO content; these observations are explored concerning the structural role of Mg^2+^ in the glass network.

## 2. Materials and Methods

### 2.1. Glass Preparation

Sodium–calcium–phosphate oxide glasses doped with magnesium were synthesized. The target glasses compositions of 10Na_2_O–60P_2_O_5_–30CaO, 5Na_2_O–5MgO–60P_2_O_5_–30CaO, 3Na_2_O–7MgO–60P_2_O_5_–30CaO and 10MgO–60P_2_O_5_–30CaO (in mol%) were prepared. The reagents: NaH_2_PO_4_ (≥99.5% Sigma Aldrich Co., St. Louis, MO, USA), NH_6_PO_4_ (99 +% ACROS ORGANICS, Geel, Belgium), CaCO_3_ (99.9 +% ChemPUR GmbH, Karlsruhe, Germany), and MgCO_3_ (extra pure ACROS ORGANICS, Geel, Belgium) were thoroughly mixed in an agate mortar and pestle. Samples were first heat-treated at 120–200 °C for 3 h, then at 500 °C for 1 h. Samples were finally heated to 1100 °C and held for 1 h in Al_2_O_3_ crucibles in an air atmosphere. Prepared glasses were annealed at 400 °C for 5 h and cooled to 50 °C for 10 h. The obtained samples had circular shapes with diameters from 10 to 15 mm and thicknesses from 2 to 2.8 mm.

### 2.2. Glass Characterization

Room-temperature powder X-ray diffraction (PXRD) was used to verify the amorphous nature of the prepared glasses, using a Bruker D2 PHASER diffractometer (Bruker AXS GmbH, Karlsruhe, Germany) with CuK_α_ radiation (l = 1.5406 Å) and LynxEye-XE detector (Bruker AXS GmbH, Karlsruhe, Germany). The data were collected from 10–90° 2θ over 120 min of scan time. The XRD results were background corrected.

An Olympus LEXT OLS4000 confocal scanning laser microscope (CSLM, Olympus Life Science, Hambur, Germany) was used to examine the morphology of freshly fractured samples. 3D color images were obtained using a 405 nm laser and photomultiplier detector with the maximum obtained optical magnification of 2160x.

The chemical compositions of the glasses were determined by energy-dispersive X-ray spectrometer (EDX GENESIS Apex Apollo X60 spectrometer, Mahwah, NJ, USA) analysis, using a scanning electron microscope (SEM), FEI Company Quanta FEG250 (FEI, Eindhoven, The Netherlands). SEM observations were done with a SE-ETD detector (secondary electron—Everhart–Thornley detector) using a 20 kV beam accelerating voltage and under the high vacuum (pressure 10^−4^ Pa). Samples were freshly fractured before measurements. EDX analysis was conducted on 3 different areas for each sample. The target and experimentally obtained compositions are reported in Table 1.

IR measurements were done with a Frontier FTIR spectrometer (PerkinElmer, Waltham, MA, USA). Plane-parallel plates from mixed powders of KBr and sample were prepared by milling and pressing. 64 scans of the spectra were obtained in the transmission mode in the range of 400–4000 cm^−1^ with a resolution of 4 cm^−1^. The IR band positions were estimated using the Origin software (version 8.5). The estimated error in the band position was ± 2 cm^−1^.

### 2.3. Impedance Spectroscopy Measurements

The electrical properties were studied by the impedance spectroscopy technique using a Novocontrol Concept 40 broadband dielectric spectrometer (Novocontrol, Montabaur, Germany). The electrical properties were measured in the frequency range from 10 mHz to 1 MHz in a temperature range of 373 K to 613 K, with an AC voltage of 1 V_rms_. The measurements were performed on polished and gold-coated plane parallel circular glass samples under air atmosphere. The temperature was controlled using a high-temperature Novotherm HT 1600 controller (Novocontrol, Montabaur, Germany).

### 2.4. Solubility in PBS

The biological degradation properties were determined by immersing the samples for 8 and 15 days in 10 mL of phosphate-buffered saline solution (PBS) at 37 °C. The PBS solution (11.9 mM phosphates, 137 mM sodium chloride and 2.7 mM potassium chloride) was prepared in proportion 1:10 to deionized water. The PBS has a pH of 7.4. PBS tests were estimated to observe the beginning of the biosolubility process in all tested samples. For each measurement, 2 samples of similar weight and size were selected from each composition. After immersion, the samples were cleaned in deionized water, dried in a desiccator for 24 h and weighed. The top layer of samples was examined with a confocal microscope and SEM. The composition of the glass surface was determined by EDX analysis.

The total percentage change in weight of soaked specimens was calculated using the following equation:

(1) $$\%\;weight\;change=\frac{final\;weight-initial\;weight}{initial\;weight}\times 100$$

### 2.5. Thermal Analysis Measurements

Glass transition (*T_g_*) and crystallization (*T_cr_*) temperatures were measured on powdered samples placed in the Al_2_O_3_ crucibles, using differential thermal analysis (DTA) up to 1000 °C in flowing nitrogen with a NETZSCH STA 409PC instrument (NETZSCH, Selb, Germany) and a heating rate of 20 °C min^−1^. The glass transition temperature was estimated based on the onset of an endothermic drift on the DTA signal. The exothermic maxima found in all samples were assigned to crystallization processes. Proteus software (version 6, NETZSCH) provided by NETZSCH was used for the estimation of thermal properties parameters with a precision of ±2%.

## 3. Results and Discussion

### 3.1. Confocal Microscopy and Compositional Analysis

**Figure 1:**
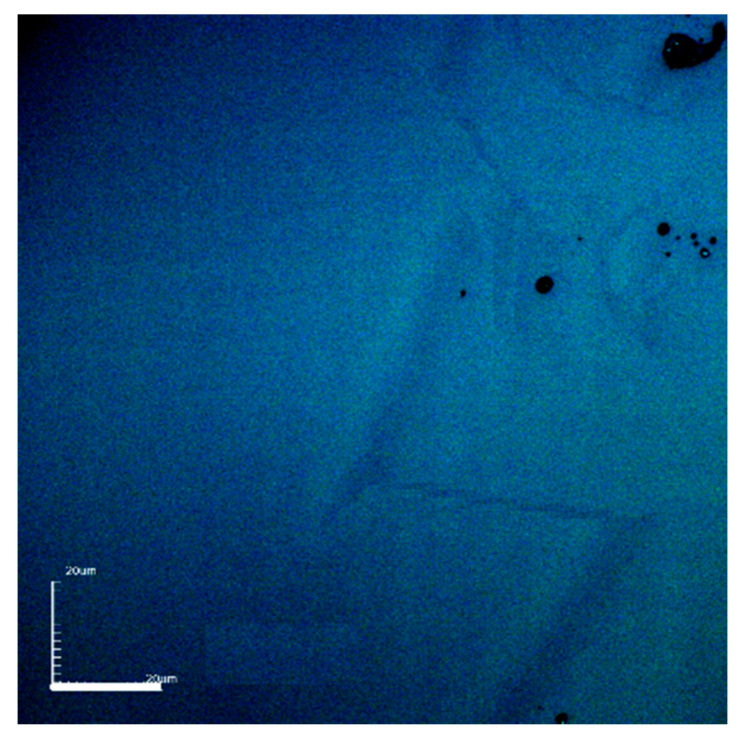
CSLM micrograph for sample 5Mg (see Table 1 for details). Scale bar is 20 μm.

Four transparent calcium–phosphate glasses with different sodium and magnesium oxide contents were synthesized. Starting and analyzed glass compositions are listed in Table 1. The samples were labeled based on the xMg content in mol% (x = 0, 5, 7 or 10). An example CSLM image for glass sample 5Mg (with 5 mol% of Na_2_O and 5 mol% of MgO) morphology is shown in Figure 1. It was typical for homogenous and amorphous materials. The morphology of other samples was similar. EDX was used to study the chemical composition of bulk samples and check Al impurities originating from the crucible material. The target and analyzed compositions were in good agreement. However, in all glasses, aluminum was also found and is included in the measured compositions (Table 1). The lowest amount of Al_2_O_3_ (~1.7 mol%) was observed in the 10Mg glass without sodium ions. Doping with Al_2_O_3_ (up to 1.5 mol%) to bioactive silicate-based glasses did not have a strong effect on their bioactive properties. At the same time, it improved their long-term stability [26]. The suitable stability is highly important for use bioactive glasses as bone implants; therefore, adding Al^3+^ ions can be even an advantage in phosphate-based glasses, which are known to react rapidly in aqueous solutions [27].

### 3.2. Structural Analysis

**Figure 2:**
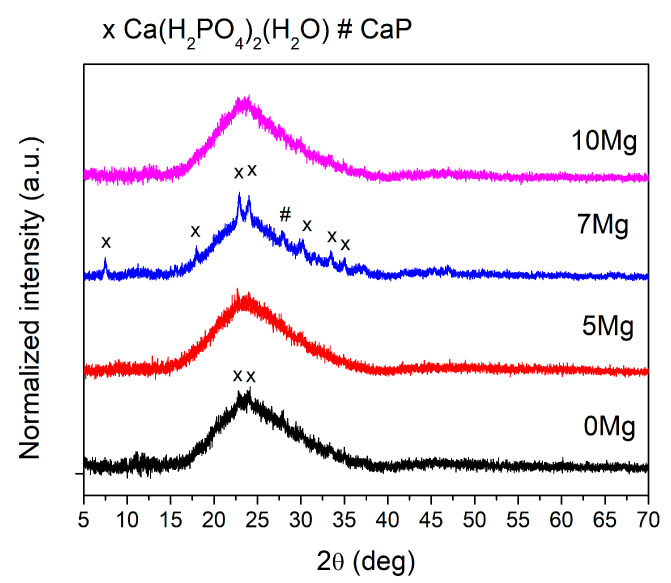
XRD curves for all as-quenched samples.

The X-ray powder diffraction was used to verify the amorphous nature of the prepared glasses. The XRD curves (Figure 2) show a typical glass bump in the range 20°–35°, which is characteristic of glasses. Samples exhibit not only the amorphous halo, but their XRD curves also show few broad reflections of low-intensity correlated with different crystalline phases. The most clearly visible reflections occurred in the sample 7Mg, which may indicate the highest content of nanocrystallites. However, in samples 0Mg, 5Mg, and 10Mg, reflections were not clearly noticeable and distinguished from halo. They may be a measuring noise. The best matching reflections fit indicated that observed nanocrystallites were mostly made from various calcium phosphates. Due to the content of the crystalline phase, the sample 7Mg can especially be referred to as a glass–ceramic nanocomposite.

**Figure 3:**
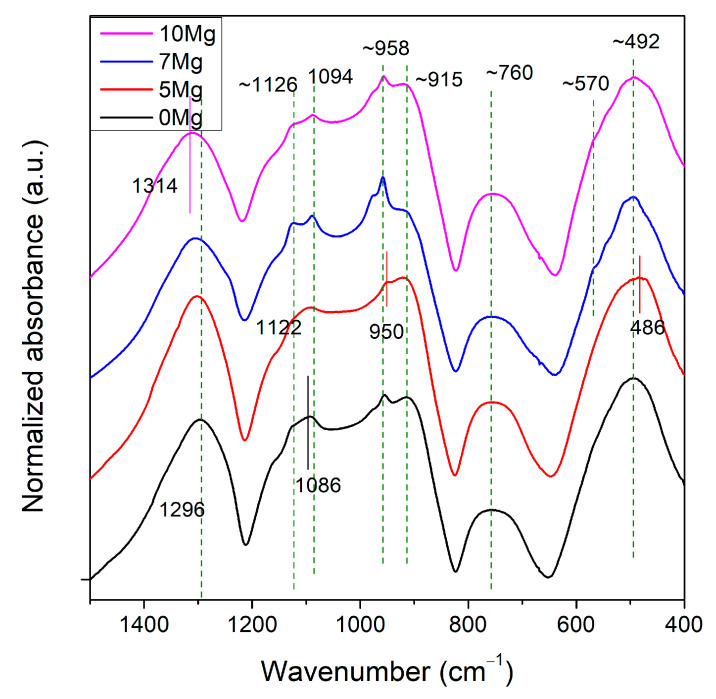
FTIR spectra for all samples.

FTIR spectra for all samples are shown in Figure 2. All materials showed mostly rounded shapes of curve bands, typical for amorphous materials. However, sample 7Mg contained small and sharpened peaks, which indicated the presence of nanocrystallites. The FTIR bands observed for all glasses are listed in Table 2 and are indicated in Figure 3.

Each composition had an O:P ratio (i.e., degree of phosphate network polymerization) of ~2.9, which is relatively high. The phosphate tetrahedra in the samples consisted mostly of Q^2^ units (polymer-like metaphosphate chains PO_2_^−^) and a small content of Q^3^ units (vitreous P_2_O_5_). Two dominating bands appeared at ~1300 and 490 cm^−1^. According to the literature [28,29,30], they were due to the asymmetric stretching vibration of Q^2^ unit (PO_2_^−^), and bending vibrations of O–P–O units, δ(PO_2_) modes of (PO_2_^−^)_n_ chain group, respectively. Bands at ~1120, 910 and 760 cm^−1^ can be correlated with symmetric stretching vibration of Q^2^ unit, (PO_2_^−^), asymmetric stretching modes of P–O–P bridges in the chain, *_ν__as_*(P–O–P), and symmetric stretching modes of P–O–P chains, vs. (P–O–P) [30,31,32,33], respectively. Additionally, in all samples, bands at ~1090 and 958 cm^−1^ were found, which are characteristic for vibrations in (PO_4_)^3−^ (Q^0^) [34] and (PO_3_)^2−^ (Q^1^) units [35]. Additionally, samples 7Mg and 10Mg show a weak signal at ~570 cm^−1^ [36], which is characteristic for nanometer-sized crystallites consisted of apatitic PO_4_^3-^ groups [37]. These results were in agreement with the XRD analysis presented earlier.

Comparing the band positions of all glasses, it can be seen that the main band ~1300 cm^−1^ characteristic for dominant Q^2^ unit shifted towards higher wavenumbers with increased the content of Mg ions in samples’ compositions (from 1296 cm^−1^ for 0Mg to 1314 cm^−1^ for 10Mg). This shift indicates a decreased number of Q^3^ units in the glass structure associated with the replacement of monovalent Na^+^ ions by divalent Mg^2+,^ having a different effect on the structure of phosphate glasses. This suggests that the depolymerization of 0Mg was higher than that of 10Mg. However, the FTIR spectra of all glasses showed only minor changes as a function of composition, which is in line with reference [4] for CaO substitution by MgO. The opposite behavior was found for the substitution of MgO for P_2_O_5,_ which increased the depolymerization of Na_2_O–CaO–MgO–P_2_O_5_ glasses [37]. It should be noted that the FTIR bands of sample 5Mg seemed to be slightly out of the behavior trend of the rest of the samples, e.g., *νs*(PO_2_)^−^, *vas*(P–O–P) in chains, *v*(O–P–O) in (PO_2_)^−^ modes, vas(P–O–P) in large in rings. This sample contained the highest amount of Al_2_O_3_. Therefore, we suspect that not only MgO but also Al_2_O_3_ influenced the structure of tested materials.

### 3.3. Electrical Properties

**Figure 4:**
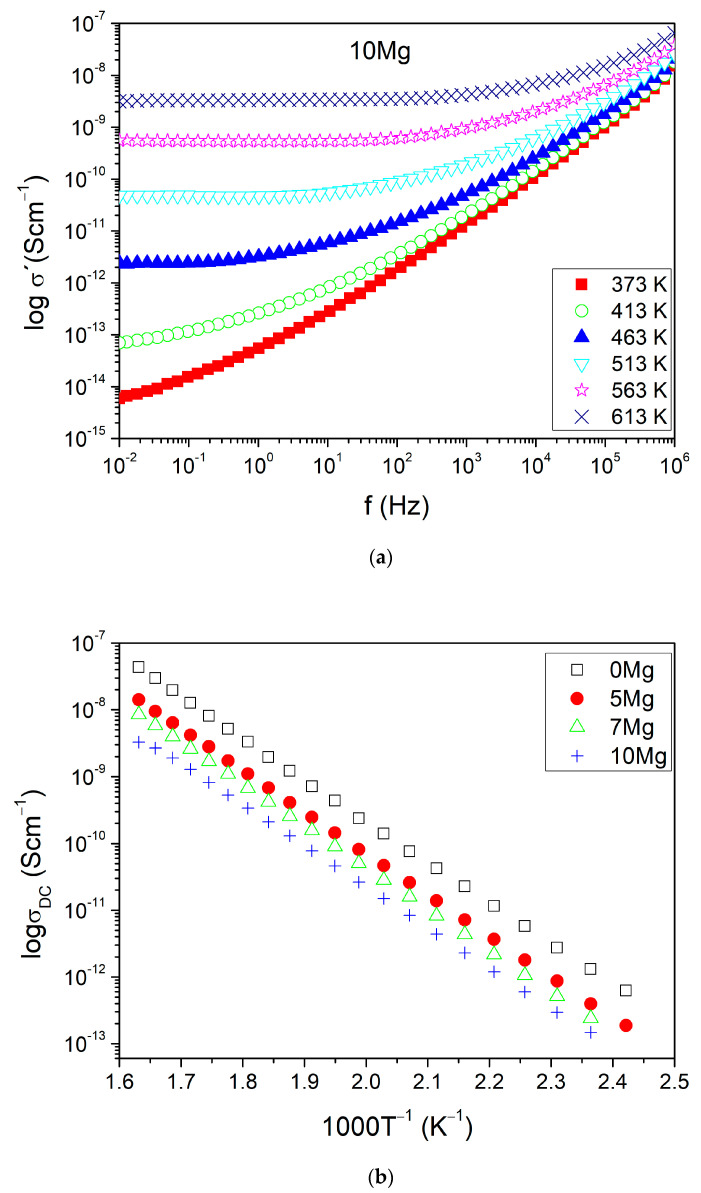
(**a**) The real part of AC conductivity versus frequency presented for different temperatures for sample 10Mg and (**b**) DC conductivity versus reciprocal temperature for all samples. The symbol size included error bars.

Figure 4a shows the real part of conductivity *σ*′ versus frequency for different temperatures for exemplary sample 10Mg. The other samples presented similar behavior to their *σ*′ spectra. The basic electrical parameters were analyzed by using Jonscher power-law [38]:

*σ*′(*ω*) = *σ_DC_*(*T*) + *A*(*T*)*ω*^*S*(*T*)^ (2)

where *σ′*(*ω*) is the real part of conductivity dependent on frequency and *σ_DC_* is the direct current (DC) conductivity, independent of frequency. Part *Aω^s^* describes alternating current (AC) dispersion. The conductivity curves contain two parts: DC conductivity (*σ_DC_*) and AC conductivity. The second part linearly increases with frequency (power-law behavior). The range of frequency for which the DC plateau occurs increases with the temperature for all tested glasses.

The *σ_DC_* values were numerically determined using Equation (2) and Figure 4a and are shown in Figure 4b for all samples. They fulfill Arrhenius’ law, which is given by the following relation:

(3) $$\sigma_{DC}T=\sigma_{0}\exp{\left(-\frac{E_{A}}{kT}\right)}^{}$$

The *σ*_0_ is the pre-exponential factor of conductivity, *E_A_* is the activation energy of mobile ions diffusion in long-range, and *k* is Boltzmann’s constant. The *lnσ*_0_ and *E_A_* values estimated from fitting (Figure 4a with Equation (3)) and the values of *σ_DC_* (evaluated at temperature 473 K) are presented in Table 3. The *σ_DC_* and *lnσ*_0_ decreased with the substitution of Na^+^ ions by Mg^2+^ ions. However, the activation energy estimated for the DC conduction process was similar for all samples. Its magnitude (~1.27 eV) was typical for an ion hopping mechanism. In that situation, the observed changes in DC conductivity were mostly due to changes of parameter *σ*_0_.

The DC conduction mechanism in sample 0Mg was due to thermally activated Na^+^ ion hopping. In sample 10Mg, the ionic conduction mechanism may have been connected with Mg^2+^ ion hopping observed in MgO–P_2_O_5_ [19,20] and ZnO–MgO–P_2_O_5_ [21] systems. The drop of DC conductivity observed between samples 0Mg (Na_2_O ~7.7 mol%, Na ~3 at %) and 10Mg (MgO 9.4 mol%, Mg ~2 at %) after total ion exchange was of one order of magnitude. This decrease can be assigned to lower mobility and content of magnesium ions in the 10Mg sample as compared to sodium ions found in the 0Mg sample. Additionally, it was shown in [21] that the increase in MgO quantity in phosphate glass may decrease its *σ_DC_*. At the same time, part of the Mg^2+^ cations may act as glass-formers, and as a result, the content of relatively free mobile ions (Mg^2+^) was reduced. Therefore, most probably in our 10Mg sample, the part of Mg^2+^ carriers did not take part in the conduction process. In the glasses containing both Na^+^ and Mg^2+^ ions, samples 5Mg and 7Mg may exhibit mixed ion hopping.

It is interesting to see whether similar activation energy was found for all samples followed a “classical” strong electrolyte model concerning ion conductors, as proposed by Anderson and Stuart [39]. Its main idea is that a mobile ion hops from one site to another and passes through a “doorway”, which opens as it passes through. Cations sites require only the presence of non-bridging oxygens. In this model, the activation energy of conduction is a sum of two parts (Equation (4)): the electrostatic binding energy of the original site *E_b_*, and the strain energy, *E_s_*, required to move an ion from one site to another [40]:

(4) $$E_{A}\left(\sigma \right)=E_{b}+E_{s},\;\mathrm{where}\;E_{b}=\frac{\beta zz_{0}e^{2}}{\gamma \left(r_{M}+r_{0}\right)}\;\mathrm{and}\;E_{s}=4\pi Gr_{D}{\left(r_{M}-r_{D}\right)}^{2}$$

Here *z* and *z*_0_ are the charges on the mobile ion and the fixed counterion—in this case, sodium and/or magnesium and oxygen with ionic radii *r_M_* and *r_O_*, respectively, *e* is the electronic charge, and *r_D_* is the effective radius of the (unopened) doorway. Parameter *G* is an elastic modulus, *β* is a “Madelung” constant, and *γ* is a covalence parameter, which indicates the degree of charge neutralization between the ion and its immediate neighbors [41].

Comparing two samples with maximal and minimal content of MgO, we observed (on FTIR results) that the 0Mg glass exhibited higher depolymerization of structure and ionic radius of mobile ions (r_Na+_ = 0.95 Å [41]) than the 10Mg glass (r_Mg2+_ = 0.72 Å). Moreover, it was shown in [42] that elastic modulus is also slightly higher for phosphate glasses doped with alkali ions than with magnesium ions. Therefore, we can assume that the strain energy part of activation energy should be higher for the hopping process of Na^+^ ions than for Mg^2+^ ions. However, while considering the electrostatic binding energy part of activation energy, the situation should be the opposite. The value of the electrostatic charge was higher for Mg^2+^ than for Na^+^. Assuming that the β parameter can be approximated by the relation: [39,41,43]: $\beta =\frac{2.1-r_{Na}}{3.5}$, the general estimation shows that it was also higher in the 10Mg glass (0.39 Å) than in the 0Mg (0.33 Å). Therefore, we can assume that the observed similar activation energy of the DC conduction process in both samples may result from the compensation of two energy parts. For alkali–alkaline glasses, the contribution of electrostatic binding energy and strain energy changes inversely with the content of Na and Mg ions in sample compositions.

**Figure 5:**
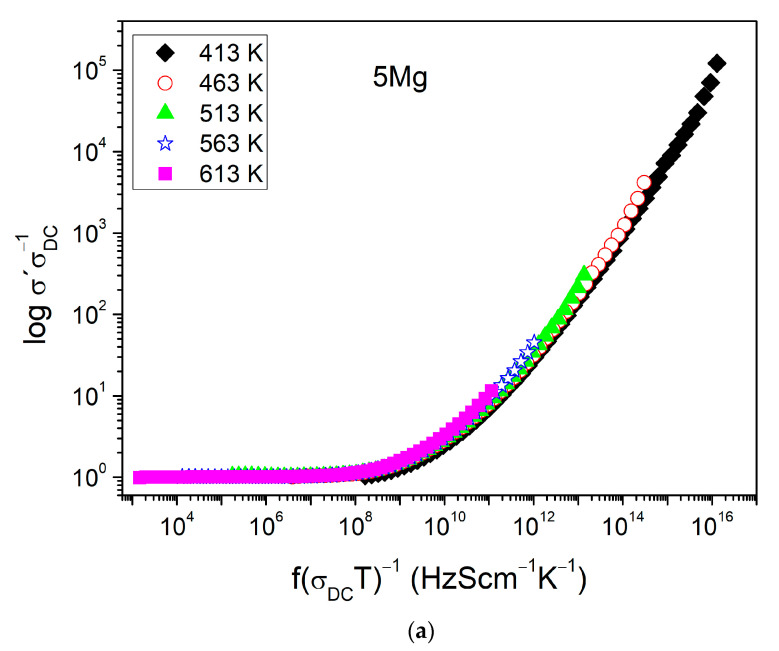

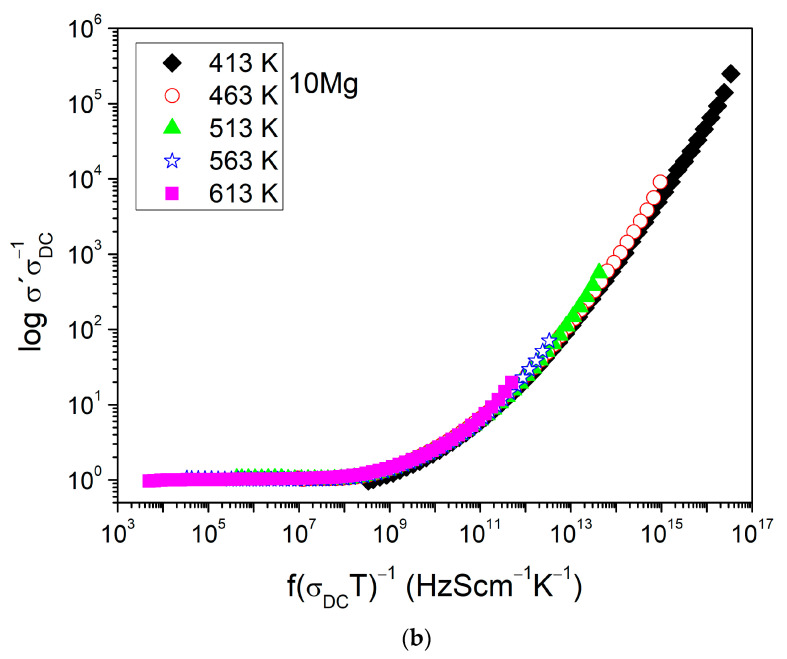
Master curves of samples: (**a**) 5Mg and (**b**) 10Mg.

In many single-ionic amorphous conductors, the total AC conductivity complies time–temperature superposition prepared. For these glasses, a master curve can be constructed as the shape of *σ*′ (*ω*) in log–log plot using Summerfield scaling [17,44]. Figure 5a,b show the master curves of *σ*′ (*ω*) constructed for samples: 5Mg and 10Mg, respectively. For both samples, the slope of *d logσ*′/*d logν* continuously increases with frequency and, as typical for glasses, is tending towards 1.0. Nevertheless, the shape of master curves of *σ*′ (*ω*) dependents on temperature not only for the mixed alkali–alkaline earth sample 5Mg (Figure 5a) but also for the alkali-free sample 10Mg (Figure 5b). The same behavior is observed for other samples. In mixed alkali–alkaline earth samples 5Mg and 7Mg, it can be correlated to different mobility of the two ionic species: Na^+^ and Mg^2+^. In this situation, an ionic transport is dominated by the ionic species with higher mobility—Na^+^ [45,46]. However, the AC conductivity of the 10Mg and 0Mg samples, which contained only one ionic species (Mg^2+^ or Na^+^), also did not obey time–temperature superposition. This indicates the presence of an additional conduction mechanism. Since all glasses are highly hygroscopic and may contain water bonds, the additional residual mechanism associated with proton hopping is possible. This process has been observed, for instance, in Na_2_O–FeO–P_2_O_5_ and CaO–FeO–P_2_O_5_ glasses [15].

### 3.4. Biosolubility in PBS

**Figure 6:**
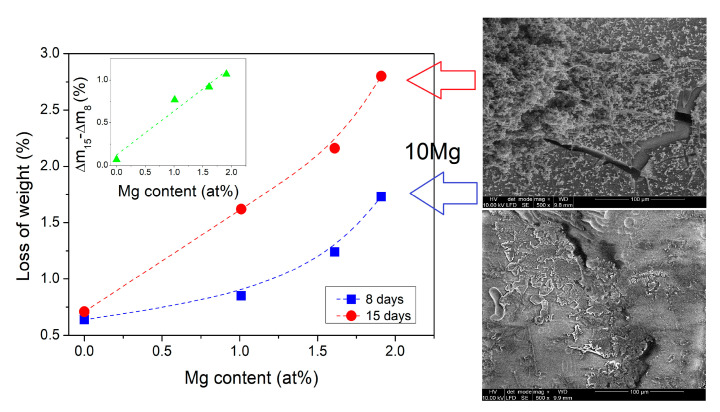
The results of the biosolubility test for all samples after 8 and 15 days of incubation in PBS as a function of Mg content. Figure insert shows the effect of Mg content on the difference between loss of weight after 15 and 8 days. On the right, the SEM micrographs of the 10Mg glass are shown, correlated by arrows with specific weight loss.

The results of the degradation test in PBS are shown as a function of Mg content in Figure 6. It can be seen that the solubility of glass samples increased with an increase in Mg content. The opposite effect of weight loss was found for the increase in Na content. The weight loss showed high dependence on Mg content. Weight loss after 15 days of incubation was approximately two times higher than after 8 days. The insert in Figure 6 shows that the difference between weight loss after 15 and 8 days was approximately linearly dependent on Mg content. The results indicate that magnesium addition advanced the biodegradation process of calcium–phosphate glasses while the glass containing only sodium ions exhibited higher stability. The observed changes in dissolution rate affected by Na_2_O substitution contradicted those presented by Lee et al. [4] for CaO substitution by MgO. They stated that the increase in MgO content decreases the degradation rate of the phosphate glass systems in the water. In contrast, in our samples, the results showed an opposite behavior for a PBS solution. Comparing the compositions, in our samples, the content of sodium ions was significantly lower (two times). At the same time, the number of phosphorous ions was higher than reported by Lee et al. [4]. Based on these findings, it could be seen that the magnesium doping effect was highly correlated with the relative contents of sodium, calcium and phosphorous. Moreover, the higher dissolution rate of tested here sodium-free glass (10Mg) could also be correlated to the chemical composition of the dissolving solution. It is known that sodium is more soluble in water than magnesium. However, PBS already contains a high concentration of sodium ions. Therefore, diffusion of magnesium to the PBS was more preferable to sodium.

**Figure 7:**
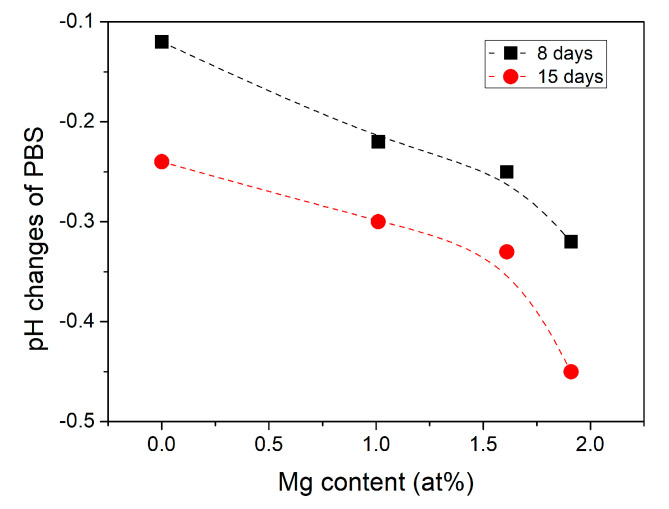
The difference in pH of the PBS after 8 and 15 days of glasses immersion as a function of Mg content. The difference was calculated as pH of the PBS after incubation of glass—pH of the PBS before incubation.

The pH of the PBS solutions was measured before and after the 8 and 15 days of incubation. The solution’s pH slightly decreased from 7.4 to approximately 7.2 after 8 days of incubation of sodium-containing glasses. In contrast, the sodium-free glass solution pH reached ~7.1. After 15 days of immersion, the pH of the solution further decreased for all glasses. The 10Mg glass, it achieved a pH value below 7. Figure 7 shows the difference in pH values of the PBS solutions before and after 8 and 15 days of incubation of glasses. The absolute values of changes in the PBS pH were higher after 15 days of incubation than after 8 days. They increased with the increase in Mg content. As described in [47], the dissolution process of the phosphate-based glasses in an aqueous solution can be divided into two steps: a hydration process and a breakage process. In the hydration process, a hydrated layer is formed on the glass surface due to Na^+^ and H^+^ ion exchange. In the breakage process, the continuous attack of water results in breaking up P–O–P bonds and breaking the glass network, releasing [PO_4_] units. The decrease in pH of solution due to dissolution of phosphate-based glasses can be related to the hydrated phosphate chains dissociating [4]. The phosphorus cations released from the breakage of P–O–P bonds tend to bond with protons to form phosphoric acid [47]. However, only the dissolution of sodium-free 10Mg glass caused the acidic pH of the PBS solution, while sodium-doped glasses only slightly acidified the solution related to the dissolution of Na^+^ ions into the solution, which increases the pH of the solution [4]. Moreover, an increase of solution pH may also be correlated with the release of Ca^2+^ and Mg^2+^ ions into the solution and chelation with the released phosphate species [47].

The decrease in solution pH observed for tested samples was significantly lower than the one described for similar glasses with higher sodium content [47] and doped with MgO [4]. Moreover, incorporating MgO resulted in increasing the rate of hydration and the rate of breakdown of the phosphate bonds. Consequently, the dissolution rate increased, and the solution pH decreased, contrary to findings in [4]. Therefore, we suppose that in the case of our glasses, the dissolution process was dominated by the releasing of phosphorus and calcium cations into the PBS (PBS lacks Ca^2+^ ions) and phosphate species chelating with released Ca^2+^. Moreover, the release of Mg^2+^ ions was more favorable than releasing Na^+^ ions due to the lack of Mg^2+^ ions in the PBS. Accordingly, phosphate species chelating with released Mg^2+^ was larger because of their higher valency state than of Na^+^. In this case, the layer built up of phosphates should occur on the glasses surfaces, as shown in Figure 6 for the 10Mg glass. The layer grew mostly from phosphorous and calcium ions, which were released from the glass surface; therefore, the higher the loss of weight was, the faster layer growth was, as shown in Figure 6 after 8 and 15 days of incubation time. It should be noted that Al needs an acidic pH to be released from glass [48]; therefore, we supposed that no Al leached into the PBS during immersion.

**Figure 8:**
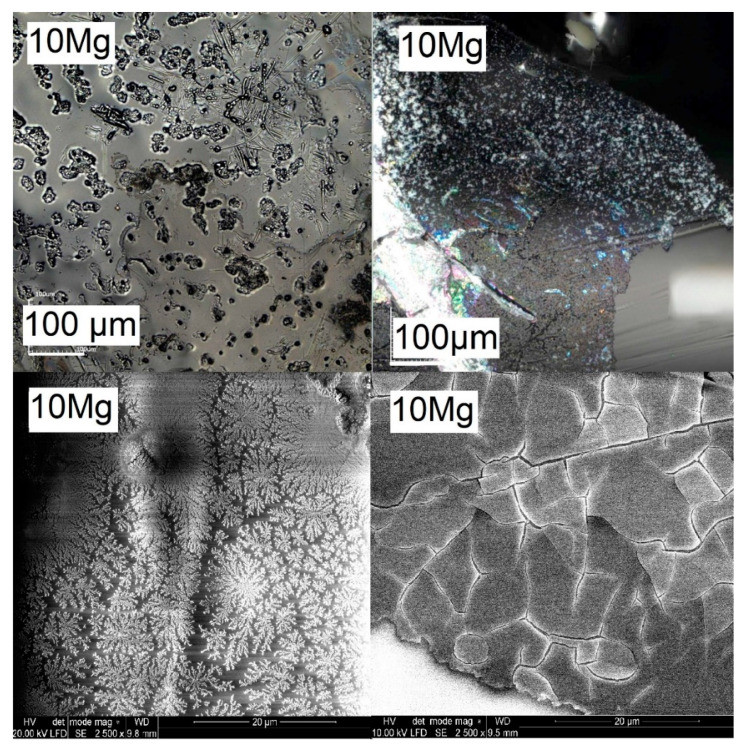
Confocal microscope (**top**) and SEM (**bottom**) micrographs after 8 (**left**) and 15 (**right**) days of incubation in the PBS for glass 10Mg.

**Figure 9:**
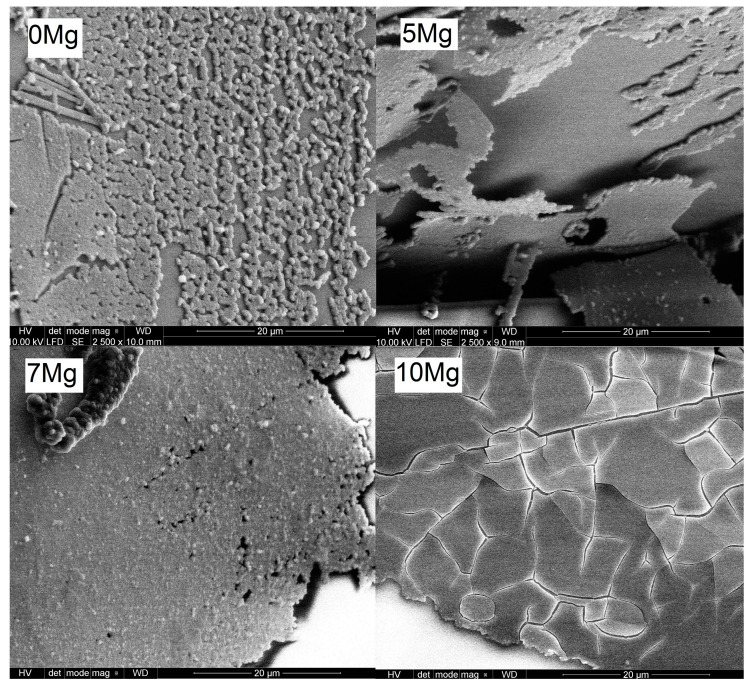
SEM micrographs after 15 days of incubation in the PBS for all glasses.

To study the effect of incubation time in PBS on the surface changes, confocal microscope and SEM micrographs of sample 10Mg are juxtaposed in Figure 8. It can be seen that 8 days of incubation was enough to observe the beginning of the nucleation process of the layer. In comparison, after 15 days, the layer was visible on most of the glass surface. The cracks observable on the layer were a consequence of the drying process. Figure 9 displays the SEM micrographs of all samples after 15 days of incubation. For all samples, the layer fragments were observed already after 8 days of incubation. After 15 days, the amount, size and thickness of the layer were higher. It should be noticed that glasses were not polished or powdered for biosolubility tests as it is often done [49,50,51]. Nevertheless, a layer built up. However, the layer mostly formed on the cracks, breakdowns and edges where the roughness of the surface was high. The SEM micrographs showed that the layer peels off the surface (Figure 9 for 5Mg glass). This peeling process was observed for samples containing Na ions, while for sample 10 Mg (Na free), no peeling was found. Moreover, the layer was the thickest on the surface of the 10Mg glass. Accordingly, we can say that glass which more dissolved (10Mg) had a larger surface area. Therefore, the layer adhesion was greater for it. A similar effect of MgO incorporation on the support of CaP-rich layer formation was found for phosphate–silicate glasses immersed in SBF [52].

**Figure 10:**
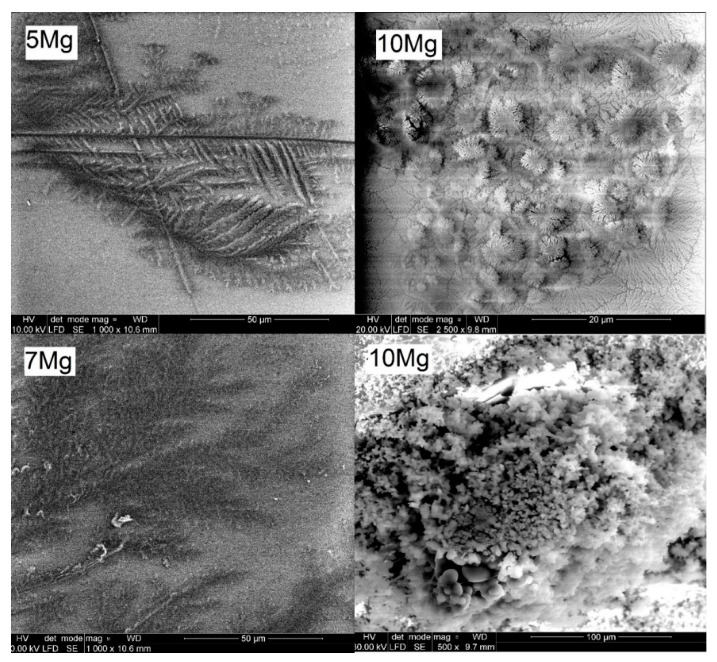
SEM micrographs after 8 (**top**) and 15 (**bottom**) days of incubation in the PBS for exemplar glasses.

Additionally, fern-like crystals were observed on the surfaces of all magnesium-doped glasses. Sample SEM micrographs of fern-like crystals are displayed in Figure 10 (left). Fern-like crystals were clearly visible after 8 days of immersion. They were most likely formed by the deposition of released magnesium and phosphate ions, as they were not found for the Mg-free 10Na glass. Figure 10 (right) also shows the spherical aggregates detected on the 10Mg glass surface after 8 (top) and 15 (bottom) days of incubation, which may have been some kind of precipitated Ca-phosphate species.

The elemental composition was checked by EDX for all samples after 8 and 15 days of incubation in the PBS. EDX analysis was done for selected areas on layers and for placed without visible layer (glass matrix). In all samples, an increase in Ca and P content was detected for both layer and glass matrix after 8 days of incubation in the PBS. Slight changes in the content of the other elements (Na, Mg and Al) were within the EDX equipment error. EDX analysis after 15 days of immersion also did not show any significant changes in the composition of all the glasses matrices, suggesting that all samples dissolved in the PBS fluid evenly. The even dissolution of biomaterial is its great advantage because it does not lose its properties, e.g., through local depletion in any of the elements. In addition, from a biological point of view, it is beneficial for material to dissolve in a controlled manner, as here, because a local excess of any element can cause toxic reactions around an implant.

EDX measurements completed after 15 days of immersion on layers showed significant changes, especially for glasses 7Mg and 10Mg. The content of P was found to be two times lower than for the glass matrix without layer. The content of Al was also significantly higher (up to 7 at %). This may suggest that during dissolution, the phosphorous and calcium ions were released into the PBS and then chelated and formed CaP-rich crystals with Al, which remained on the glass surface. Apart from apatite formation, several other possible phases of calcium orthophosphates may be formed in the PBS. However, at the later stages, all of them will convert into HA in various pathways. To confirm the possible presence of hydroxyapatite (Ca_10_(PO_4_)_6_(OH)_2_]) layer created during immersion or indicate the higher potential for HAp creation on the sample surface, the Ca/P ratio was studied. For silicate-based glass-ceramics, this value was close to 1.69 (in at %) [48,53], similar to HAp. For all tested glasses, the Ca/P ratio varied between 0.26 and 0.29 and slightly increased after immersion. The highest Ca/P ratio ~0.47 was found for the spherical aggregates observed on 10Mg glass (Figure 9 down, right) and layer on glass 7Mg; ~0.42, after 15 days of immersion. Both results suggest the presence of Ca-phosphates. However, values are lower than the theoretical of HAp, which may have resulted from a too-small thickness of the layer and the EDX signal as measured both on the layer and the glass matrix below.

### 3.5. Thermal Properties

**Figure 11:**
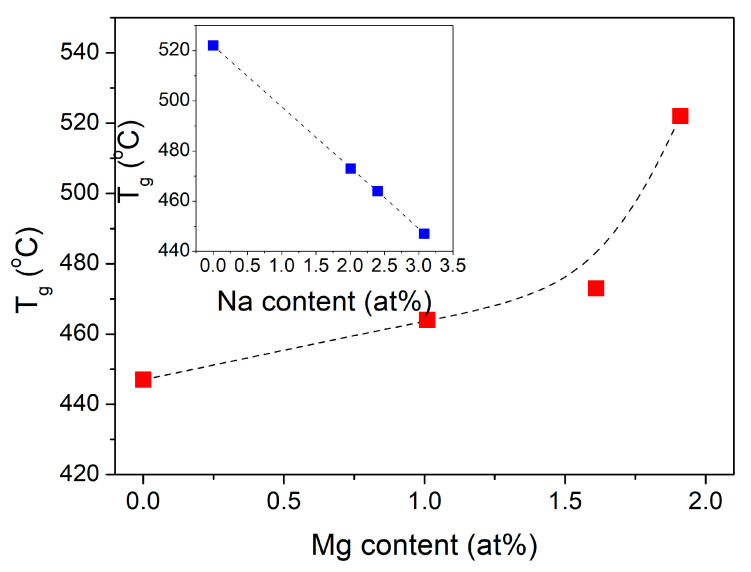
*T_g_* as a function of Mg content and in insert function of Na content. The experimental errors are about the same size as the symbols.

All samples clearly showed the glass transition temperature and one broad exothermic process. The glass transition and crystallization temperatures increased with the magnesium addition. *T_g_* increased for 75 degrees, from 447 °C for sample 0Mg to 522 °C for sample 10Mg (see Table 4). Figure 11 presents the *T_g_* versus sodium and magnesium content. It can be seen that *T_g_* decreased linearly with increased Na content. This may have been due to the different structural roles of sodium and magnesium ions in the phosphate matrix. It is known that adding modifier ions like Ca^2+^ and Mg^2+^ [54] results in depolymerization of the glass network and decreases the glass *T_g_*. When high field strength Mg^2+^ ions substitute low field strength ions: Ca^2+^ or Na^+^, the *T_g_* of phosphate glass increases. Furthermore, adding Al_2_O_3_ depolymerizes the phosphate network but increases the *T_g_* is due to the strong P–O–Al crosslinks [55,56].

These results were also in agreement with the FTIR findings, which showed that the polymerization of the phosphate network increased with the increase in MgO content. They also followed the literature data [4]. However, our samples exhibited higher *T_g_* values than the corresponding glasses with higher Na_2_O (20 mol%) and lower P_2_O_5_ (50 mol%) contents, even for glasses doped with 30 mol% MgO.

The bioglass resistance to the crystallization process during heating is frequently estimated as an important parameter for its practical use. Glass thermal stability is generally defined as the difference between the onsets values of the first crystallization process and the glass transition temperatures *S = T_exo1, onset_* − *T_g_* [57]. Table 4 presents glass thermal stability values calculated for all samples. It can be seen that glass thermal stability decreased with increased Mg content from 199 °C for sample 0Mg to 175 °C for 10Mg. The decrease in the glass thermal stability due to increased MgO content was a consequence of slower shiftiness of the beginning of the crystallization process than the one observed for *T_g_*. This may suggest that the doping with MgO slightly increases the glass tendency to crystallize. However, in the case of silicate-based glasses, a similar tendency was not observed [58].

## 4. Conclusions

In the current paper, we studied increasing Mg content by replacing Na and its effect on the properties. Four sodium–calcium–phosphate glasses substituted with different content of magnesium oxide were prepared. Most of the glasses were X-ray amorphous. IR spectroscopy analysis showed a high degree of spectral overlap between the studied glassy materials. All of them had phosphate networks made up mostly of Q^2^ and small content of Q^3^ phosphate units. Additionally, in all samples, bands correlated with Q^0^ and Q^1^ groups were detected, which suggested the presence of (PO_4_)^3−^ and (PO_3_)^2−^ groups. With adding Mg, the network was increasingly polymerized, which was reflected in decreased DC conductivity, increased biosolubility and glass transition temperature. The DC conductivity values decreased by one order of magnitude from 4.21 × 10^−11^ to 4.21 × 10^−12^ Scm^−1^ after substitution of MgO for Na_2_O in the Na_2_O–CaO–P_2_O_5_ glassy system. In contrast, the activation energy of the DC conduction process remained close to ~1.27 eV for all samples. The behavior of AC conductivity indicated the presence of at least two conduction mechanisms for all samples. In mixed alkali–alkaline earth samples, the conduction mechanism was associated with hopping of two different ionic species: Na^+^ and Mg^2+^. However, for all samples, an additional residual mechanism was also possible: proton hopping. Tests in PBS showed that all prepared samples exhibited biosolubility properties and evenly dissolved at an appropriate rate in PBS. Furthermore, the magnesium addition enhanced the biosolubility of calcium–phosphate-based bioglasses by increasing their dissolution rate and supporting forming CaP-rich layers on the surface. The results found for the biodegradation process present new insights into the role played by MgO that contradict available literature data on phosphate bioglasses. The transition temperature increased by up to 75 °C for Na_2_O–CaO–P_2_O_5_ glass after substitution of MgO for Na_2_O. The high increase in *T_g_* was caused by the replacement of low field strength ions Na^+^ by high field strength Mg^2+^ ions and by the increase in the polymerization of the network. Presented materials could be a possible candidate for bone implant application.

## Figures and Tables

**Table 1:** IDs, starting and final compositions of all samples.

ID	Starting Composition (mol%)	Final Composition (mol%)
0Mg	10Na_2_O–60P_2_O_5_–30CaO	7.7Na_2_O–56.9P_2_O_5_–32.6CaO–2.7Al_2_O_3_
5Mg	5Na_2_O–5MgO–60P_2_O_5_–30CaO	6Na_2_O–5MgO–55.5P_2_O_5_–29.8CaO–3.7Al_2_O_3_
7Mg	3Na_2_O–7MgO–60P_2_O_5_–30CaO	4.9Na_2_O–7.8MgO–55.8P_2_O_5_–29CaO–2.5Al_2_O_3_
10Mg	10MgO–60P_2_O_5_–30CaO	9.4MgO–57.7P_2_O_5_–31.2CaO–1.7Al_2_O_3_

**Table 2:** FTIR bands positions and their assignments (see Figure 3 and text for details), where *v*, vs. and *vas* means vibrations, symmetric stretching vibrations and asymmetric stretching vibrations, respectively.

Sample ID	*νas*(PO_2_)^−^	*νs*(PO_2_)^−^	*νas*(PO_3_)^2−^ End Groups	*vas*(P–O–P) in Large Rings	*vas*(P–O–P) in Chains	*vs*(P–O–P)	*v*(O–P–O) in (PO_2_)^−^ Modes
0Mg	1296	1126	1094	956	910	758	492
5Mg	1304	1122	1094	950	918	758	486
7Mg	1312	1126	1088	960	914	764	494
10Mg	1314	1128	1086	958	916	760	492
References	[28,29]	[28,29]	[28,29]	[28]	[28,30,31,32]	[30,31,32]	[30]

**Table 3:** Values of DC conductivity estimated at 473 K, activation energy of DC conduction process and *σ*_0_ parameter.

IDs	*σ_DC_* at 473 K (Scm^−1^)	*E_A_* (eV) ±0.001	*lnσ*_0_ (KScm^−1^) ±0.01
0Mg	4.21 × 10^−11^	1.267	13.38
5Mg	1.38 × 10^−11^	1.273	12.41
7Mg	8.22 × 10^−12^	1.276	11.99
10Mg	4.34 × 10^−12^	1.256	10.86

**Table 4:** DTA results of experimentally analyzed samples; glass transition (*T_g_*), exothermic process onset (*T_cr onset_*), crystalline peak position (*T_cr middle_*), and glass stability (*S*).

Sample ID	*T_g_* (°C)	*T_cr onset_* (°C)	*T_cr middle_* (°C)	*S* (°C)
0Mg	447	646	708	199
5Mg	464	655	735	191
7Mg	473	662	779	189
10Mg	522	697	748	175

## Data Availability

The data that support the findings of this study are available from the corresponding author upon reasonable request.
